# Circulating Amino Acid Concentration after the Consumption of Pea or Whey Proteins in Young and Older Adults Affects Protein Synthesis in C2C12 Myotubes

**DOI:** 10.3390/nu16172870

**Published:** 2024-08-27

**Authors:** Jérôme Salles, Marine Gueugneau, Karima Laleg, Christophe Giraudet, Phelipe Sanchez, Adeline Blot, Ruddy Richard, Nathalie Neveux, Catherine Lefranc-Millot, Caroline Perreau, Laetitia Guérin-Deremaux, Yves Boirie, Stéphane Walrand

**Affiliations:** 1 Unité de Nutrition Humaine (UNH), Université Clermont Auvergne, INRAE, CRNH Auvergne, 63000 Clermont-Ferrand, France; jerome.salles@inrae.fr (J.S.); marine.gueugneau@inrae.fr (M.G.); karimalaleg@gmail.com (K.L.); christophe.giraudet@inrae.fr (C.G.); phelipe.sanchez@uca.fr (P.S.); yves.boirie@inrae.fr (Y.B.); 2 CHU Clermont-Ferrand, Centre de Recherche en Nutrition Humaine Auvergne, 63000 Clermont-Ferrand, France; ablot@chu-clermontferrand.fr (A.B.); ruddy.richard@uca.fr (R.R.); 3 Service de Biochimie, Hôpital Cochin, APHP, Université Paris Centre, 75679 Paris CEDEX 14, France; nathalie.neveux@aphp.fr; 4 Life Sciences Research & Development, Nutrition & Health Department, Roquette, 62136 Lestrem, France; catherine.lefranc-millot@roquette.com (C.L.-M.); caroline.perreau@roquette.com (C.P.); laetitia.guerin-deremaux@roquette.com (L.G.-D.); 5 CHU Clermont-Ferrand, Service Nutrition Clinique, 63000 Clermont-Ferrand, France

**Keywords:** amino acids, pea protein, whey protein, aging, protein synthesis, skeletal muscle

## Abstract

As older adults tend to reduce their intake of animal-source proteins, plant-source proteins may offer valuable resources for better protein intake. The aim of this study was to assess whether the pea proteins can be used to achieve blood amino acid levels that stimulate muscle protein synthesis. We measured variations in plasma amino acid concentrations in young and older adults given pea (NUTRALYS^®^ S85 Plus) or whey proteins either alone or in a standardized meal. The effect of amino acid concentrations on protein synthesis in C2C12 myotubes was determined. In terms of results, plasma amino acid concentrations reflected the difference between the amino acid contents of whey and pea proteins. Blood leucine showed a greater increase of 91 to 130% with whey protein compared to pea protein, while the opposite was observed for arginine (A greater increase of 147 to 210% with pea compared to whey). Culture media prepared with plasmas from the human study induced age-dependent but not protein-type-dependent changes in myotube protein synthesis. In conclusion, pea and whey proteins have the same qualities in terms of their properties to maintain muscle protein synthesis. Pea proteins can be recommended for older people who do not consume enough animal-source proteins.

## 1. Introduction

In older adults, maintaining adequate protein intake is essential to preserve muscle mass and function and delay the development of sarcopenia. Sarcopenia is an important life-long process with a complex and multifactorial etiology. Among the many factors involved, an imbalance in rates of protein synthesis and breakdown can lead to a progressive loss of skeletal muscle proteins with advancing age [1]. The mechanisms involved in this dysregulation are not clearly understood. However, adequate amino acid availability is a potent stimulus for muscle protein synthesis [2], and amino acid transport to muscle cells is a major determinant in the regulation of protein anabolism [3,4]. Muscle metabolism in older people may be affected by alterations in the levels and bioavailability of different amino acids, partly driven by a higher splanchnic extraction of dietary amino acids [5]. In addition, the rate of muscle protein synthesis depends on the effects of amino acids and insulin. These effectors stimulate protein synthesis and accelerate protein turnover in this tissue. The ability of insulin and amino acids to activate muscle protein synthesis therefore partly determines muscle protein mass. Aging is characterized by a reduced muscle response to the activating effects of amino acids and insulin on protein synthesis. This phenomenon, known as anabolic resistance, contributes to the loss of skeletal muscle with age [6,7,8].

The research exploring the role of plant proteins is expanding, driven by increasing awareness of the health benefits associated with plant-based diets and the need for sustainable food production practices to support global population growth [9]. In older people, plant proteins have gained significant scientific interest due to several factors. Older people are interested in plant proteins because their appetite for protein-rich meat products diminishes with age. In addition, choosing plant-based sources of protein increases the fiber and micronutrient contents of the diet while reducing the intake of saturated fatty acids and cholesterol. Although plant proteins vary in their digestibility and amino acid profile, advancements in food processing and formulation have led to the development of plant-based protein sources with improved digestibility and amino acid balance, making them suitable for meeting the specific protein needs of older individuals [10]. Combinations of plant-based proteins can offer comparable nutritional quality to animal-source proteins while, in many cases, being lower in saturated fats and cholesterol. In addition, plant-based proteins generally have a lower environmental footprint than animal-source proteins [11]. Growing concern around the environmental impact of food production is driving increasing interest in promoting plant-based diets as a sustainable option for older adults and the general population.

While plant proteins can offer many health benefits, some may have imbalanced amino acid profiles, meaning they may lack or undersupply certain essential amino acids (EAA). Most plant proteins have a limiting amino acid. A limiting amino acid is represented by a content of one or more essential amino acids below requirements. As a result, this protein intake is not complete in relation to requirements and may slow down body protein turnover [12]. For example, most plant proteins are deficient in one or more of the EAA, such as lysine, methionine, or tryptophan, which may pose challenges for people who rely heavily on plant-based sources for their protein intake, particularly if they do not consume a diversified diet or if they have increased protein needs due to factors such as aging. As older adults may have different protein requirements and metabolic processes than younger individuals, it is crucial to examine the effects of plant proteins on amino acid profiles and muscle protein metabolism in older people in order to understand what role they can play in addressing age-related muscle loss, i.e., sarcopenia. Muscle protein synthesis and muscle protein degradation are regulated by blood amino acids, among other factors [13,14]. Amino acids, particularly EAA, serve as substrates for protein synthesis in muscle. Elevated blood levels of amino acids (e.g., after a meal or protein ingestion), especially EAA, stimulate muscle protein synthesis. The presence of EAA activates signaling pathways, such as the mammalian target of rapamycin (mTOR), which promotes protein synthesis in muscle cells [15]. Amino acid availability also affects muscle protein degradation [16]. In conditions of amino acid deficiency or fasting, the body may initiate muscle protein degradation to release amino acids from muscle tissue in order to maintain blood amino acid levels and support vital functions elsewhere in the body [17]. However, under normal dietary conditions, elevated blood amino acids inhibit muscle protein degradation. For instance, Combaret et al. have shown that a leucine-supplemented diet can restore the defective postprandial inhibition of protein degradation in skeletal muscle [16]. Therefore, in older people, adequate dietary protein intake, particularly with a sufficient amount of leucine and EAA, is crucial for maximizing muscle protein synthesis, minimizing muscle protein degradation, and thereby promoting muscle maintenance. Blood amino acids serve as key nutritional signals that regulate these processes, and so adequate protein intake is vital to maintaining muscle health [2,4]. However, we still know little about whether the post-ingestive signal, i.e., the increase in blood amino acid concentration provided by plant proteins, is sufficient to correctly regulate muscle protein metabolism in older people. A significant number of studies have shown that pea proteins are able to stimulate muscle protein synthesis in young exercisers or animals [18,19,20]. However, to date, no study has focused on variations in blood concentrations of amino acids following the consumption of pea proteins alone or in a meal in young or older individuals. Furthermore, in older people, the effect of the fluctuations in blood amino acids on the rate of protein synthesis within muscle cells has not yet been clarified.

Comparative studies between plant-source and animal-source proteins can usefully help to evaluate their differential effects on amino acid profiles and muscle protein metabolism in older adults. A deeper understanding of how plant proteins compare to animal proteins in terms of their ability to support muscle health would help to develop dietary recommendations for older individuals. Therefore, the aim of this study was to evaluate the potential of pea proteins compared to whey proteins, provided alone or in a standardized meal, to achieve a plasma amino acid profile able to stimulate muscle protein synthesis. The stimulatory action of blood amino acids on muscle protein synthesis after ingestion of pea or whey proteins was evaluated via an indirect in vitro method in C2C12 myotubes incubated with plasma from young and older subjects.

## 2. Materials and Methods

### 2.1. Subjects

Thirty healthy male subjects, including 15 young individuals (20–28 years old) and 15 older individuals (65–81 years old), were recruited to participate in the study. Participants had a body weight > 55 kg and a body mass index between 22 and 28 kg/m^2^. Volunteers did not present food allergies or contraindications to consumption of the tested products, nor pathologies or medications incompatible with the study (tumor or inflammatory disease, renal failure, malabsorption syndrome, diabetes, dyslipidemia, corticosteroid treatment, antibiotic treatment, or non-steroidal anti-inflammatory drug treatment). Subjects gave written informed consent after first being explained the nature, methodology, and possible risks of the study. Participants were recruited by the study team of the joint INRAE-Clermont Auvergne University Human Nutrition Unit in Clermont-Ferrand, France, from existing databases and through advertisements in local media. The study protocol was approved by the local institutional review board (CPP Tours-Région Centre-Ouest 1, France) and the French National Agency for the Safety of Medicines and Health Products and performed in accordance with Declaration of Helsinki principles. This trial was registered at clinicaltrials.gov under number NCT06381869.

### 2.2. Study Design

This study was a randomized crossover trial. All eligible subjects received the following four dietary treatments in a balanced, random-order sequence, with at least a 1-week washout interval between treatments: a pea protein solution alone (PP), a whey protein solution alone (WP), a standardized meal followed by a pea protein solution (MPP), and a standardized meal followed by a whey protein solution (MWP). Pea protein powder (NUTRALYS^®^S85 Plus; Roquette, Lestrem, France) and whey protein powder (Pronativ^®^ 95; Lactalis Ingredients, Bourgbarré, France) were dissolved in 300 mL of water. Each test protein solution was prepared to provide 0.41 g of protein per kg of body weight (Supplementary Table S1) [21]. The compositions of protein powders are presented in Table 1. The standardized meal consisted of red beetroot and a salad dressing, mashed potatoes with butter, and a fruit compote. The test meals represented one-third of their daily energy intake, i.e., 11 kcal/kg body weight, and had a standard nutrient composition, i.e., 15, 55, and 30% of total energy as protein, carbohydrate, and fat, respectively. Therefore, the content of each ingredient making up the meal was calculated to provide a total energy intake (meal + test protein solution) of 11 kcal/kg body weight and a total protein intake of 0.54 g/kg body weight (0.41 g/kg from the test protein solution and 0.13 g/kg from the standardized meal) [21]. As the pea protein powder contained less protein and more fat than the whey protein powder, meal composition had to be adjusted depending on the protein solution tested, as indicated in Supplementary Table S2.

**Figure 1:**
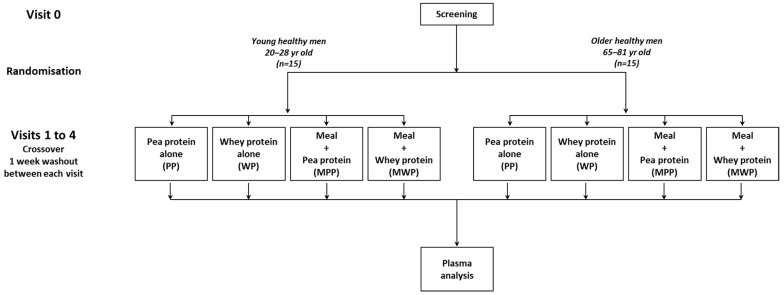
Study design.

In addition to the screening visit, participants attended the Human Nutrition Research Center in Clermont-Ferrand (France) in a fasting state on four different mornings, separated by at least one week (Figure 1). On the evening prior to each day of testing and before 21:00, subjects were asked to consume the same standardized foods in the same standardized quantities as defined by a dietician and then to fast until the next morning. On the day of testing, subjects arrived at 08:00, a catheter was placed in an antecubital vein, and a baseline blood sample was taken (T0). Participants then received one of the four dietary treatments. Standardized meals were consumed within 13 min, and test solutions were consumed within 2 min. Regular blood sampling was performed at 15, 30, 60, 90, 120, 180, 240, 300, and 360 min after the ingestion of the test protein solution. Blood samples were collected in EDTA-containing tubes and lithium heparin tubes and centrifuged at 1300× *g* for 10 min at 4 °C. A part of the heparin plasma and all of the EDTA plasma were aliquoted and stored at −80 °C until analysis. The remaining heparin plasma was deproteinized with a 30% (*w*/*v*) sulfosalicylic acid solution. After centrifugation at 5500× *g* for 5 min at 4 °C, the supernatant fractions were removed and stored at −80 °C until plasma amino acid (AA) concentration measurements.

### 2.3. Analytical Procedures

#### 2.3.1. Determination of Plasma AA Concentrations

Amino acids were separated, and concentrations were quantified by ion exchange chromatography using an amino acid autoanalyzer (Amino Tac, JLC-500/V; Jeol Ltd., Tokyo, Japan) with ninhydrin derivatization [22].

#### 2.3.2. Measurement of Plasma Biomarkers and Markers of Inflammation

Glucose, cholesterol, and triglyceride plasma concentrations were assessed at the screening visit using a Konelab 20 analyzer (Thermo-Electron Corporation, Waltham, MA, USA). Plasma insulin and markers of inflammation (TNF-α and IL-6) were measured using ELISA kits (insulin: Alpco, Eurobio Scientific, Les Ulis, France; TNF-α and IL-6: R&D Systems, Lille, France). TNF-α and IL-6 plasma concentrations were measured at T0 on each dietary treatment visit, and the mean of the four values was then calculated for each subject and used to calculate the mean of the group.

#### 2.3.3. Muscle Cell Culture

C2C12 myoblasts (ATCC, Manassas, VA, USA) were grown to 80–90% confluence in DMEM supplemented with 10% fetal calf serum at 37 °C in a 5% CO_2_-humidified atmosphere. Cells were then induced to differentiate into myotubes by switching to DMEM containing 2% heat-inactivated horse serum [23,24]. After 5 days of differentiation, C2C12 myotubes were serum- and amino acid-starved in HBSS for 2 h. Myotubes were then conditioned with HBSS containing 10% heparin plasma prepared from the human study. We compared two plasma samples from each participant at each dietary treatment: plasma samples prepared at baseline (T0) versus plasma samples at the peak plasma leucine concentration (Cmax) (to induce maximum protein synthesis in treated C2C12 myotubes).

#### 2.3.4. Measurement of Protein Synthesis and Immunoblot Analysis

To measure protein synthesis in plasma-conditioned C2C12 myotubes, we adapted the surface sensing of translation (SUnSET) method described by Goodman et al. [25]. In preliminary experiments, and in accordance with Crossland et al. [26], we observed a linear rate of puromycin incorporation in neosynthesized peptides in C2C12 cells until 4 h of incubation. Therefore, to increase puromycin labeling and sensitivity between the different plasma treatments, C2C12 myotubes were treated with plasmas for 4 h in the presence of 1 µM puromycin, then homogenized in an ice-cold lysis buffer (50 mM HEPES pH 7.4, 150 mM NaCl, 10 mM EDTA, 10 mM NaPPi, 25 mM β-glycerophosphate, 100 mM NaF, 2 mM Na orthovanadate, 10% glycerol, 1% Triton X-100) containing a protease-inhibitor cocktail (Sigma-Aldrich, Saint-Quentin-Fallavier, France) as previously described [23,24,27]. Puromycin incorporation into nascent polypeptides was measured by separating 15 µg of denatured proteins by SDS-PAGE on a polyacrylamide gel until the dye front was 1.5 cm from the bottom of the gel [23,26]. The p70 S6 kinase content and phosphorylation level were measured by loading 50 µg of denatured proteins onto a polyacrylamide gel. The SDS-PAGE-separated proteins were then transferred to a polyvinylidene membrane (Millipore, Molsheim, France) [28]. Immunoblots were blocked with TBS-Tween-20 0.1% containing 5% dry milk and then probed overnight at 4 °C with primary antibodies (mouse monoclonal anti-puromycin antibody, Millipore, Molsheim, France; anti-phospho p70 S6 kinase (Thr389) and anti-total p70 S6 kinase primary antibodies, Cell Signaling Technology, Ozyme, Saint-Quentin-en-Yvelines, France). The immunoblots were then incubated with horseradish peroxidase-conjugated secondary antibodies (swine anti-mouse secondary antibody, DAKO, Trappes, France; goat anti-rabbit secondary antibody, Thermo Fisher Scientific, Courtaboeuf, France). Luminescence visualization was performed using ECL Western Blotting Substrate (Pierce, Thermo Fisher Scientific, Courtaboeuf, France) and a Fusion Fx imaging system (Vilber Lourmat, Collegien, France). Band densities were quantified using MultiGauge 3.2 software (Fujifilm Corporation, Tokyo, Japan). Expression of the total amount of p38 was used to normalize protein loading between samples [23,29]. The p70 S6 kinase activation state was evaluated based on the ratio of phosphorylated protein to total protein expression. Results were expressed as a change in puromycin incorporation in neosynthesized peptides and a change in the p70 S6 kinase activation state in C2C12 myotubes between conditioning with human plasma prepared at baseline (T0) and human plasma prepared at peak plasma leucine concentration (Cmax). In total, the rate of p70 S6 kinase phosphorylation and level of protein synthesis were measured on 240 samples of C2C12 myotubes conditioned with human plasmas, and these measurements required 24 polyacrylamide gels and polyvinylidene membranes. As a result, it is impossible to show a gel representative of all conditions, but all uncropped and unedited blot/gel images can be provided upon request.

### 2.4. Statistical Analyses

All data were expressed as mean ± SEM. An unpaired *t*-test was used to analyze the baseline physical and biochemical characteristics of the study participants measured at the screening visit. The kinetics of plasma leucine concentration changes after dietary treatments were analyzed by 4-way repeated measures ANOVA to explore the main effects (age, protein source, type of protein intake, i.e., on its own or as part of a standardized meal, and time) and their interactions. For amino acid data, the incremental area under the curve (iAUC) was calculated by subtracting the baseline value measured at T0 from each subsequent timepoint and then applying the trapezoidal rule. To avoid negative values in the sum of the trapezoid areas, the iAUC was calculated between T0 and 180 min, except for sulfur-containing amino acids, for which the iAUC was calculated between T0 and 120 min. The AUC for glucose and insulin was calculated between T0 and 180 min. Incremental maximal concentration (iCmax) was defined as the maximum increase in plasma concentration change of an amino acid or a sum of several amino acids observed post baseline. The Cmax of glucose or insulin was defined as the peak plasma concentration. Three-way repeated-measures ANOVA was performed on iAUC, AUC, iCmax, and Cmax data and immunoblot results to explore the main effects of age, protein source, and type of protein ingestion and the interaction effects of age × protein source, age × type of protein ingestion, protein source × type of protein ingestion, and age × protein source × type of protein ingestion. A two-way repeated-measures ANOVA was performed on baseline values (basal amino acid, glucose, and insulin concentrations, basal p70 S6 kinase phosphorylation state, and basal protein synthesis rate) to explore the main effects of age and repetition and the effect of age × repetition interaction. A Tukey-Kramer post hoc test was performed to explore significant main and interaction effects. Significant main effects were analyzed on the marginal means. Differences were considered significant at *p* < 0.05. Statistical analysis was performed using NCSS 2020 software (NCSS LLC, Kaysville, UT, USA) and StatView software (version 4.02; Abacus Concepts, Berkeley, CA, USA).

## 3. Results

### 3.1. Participant Characteristics

The physical and biochemical characteristics of the subjects at screening are presented in Table 2. The younger subjects presented higher heights and weights than the older subjects (*p* < 0.05 and *p* < 0.001, respectively), but body mass index (BMI) did not differ between age groups. Fasting glucose and cholesterol plasma concentrations were significantly lower in young participants than in older participants (−9%, *p* < 0.05 and −25%, *p* < 0.001, respectively). IL-6 and TNFα were determined at T0 before each dietary treatment (Table 3). At the fasting state before dietary challenge, there was no between-group difference in plasma TNFα levels, but plasma IL-6 concentrations were significantly higher in older participants than young participants (+130%, *p* < 0.05).

### 3.2. Plasma Amino Acid Concentration Changes

#### 3.2.1. Leucine

**Figure 2:**
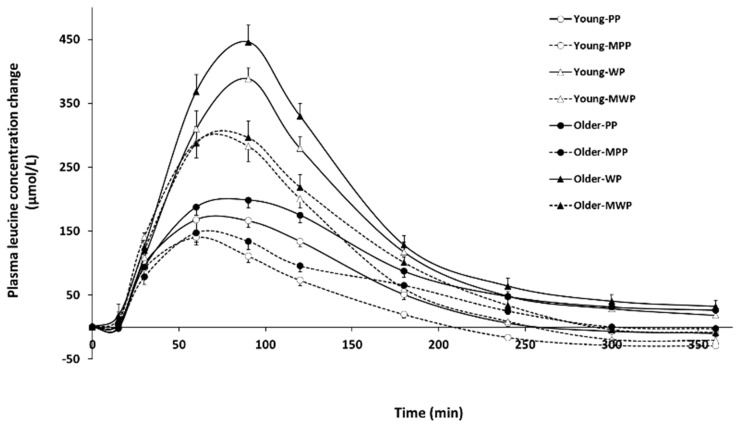
Changes in leucine concentrations in plasmas from young and older participants after the PP, WP, MPP, and MWP dietary treatments. To calculate the plasma leucine concentration change, the baseline value measured at T0 was subtracted from the other values measured at the different time-course points. Results are expressed as means ± SEM. PP: pea protein solution; WP: whey protein solution; MPP: standardized meal plus pea protein solution; MWP: standardized meal plus whey protein solution.

Figure 2 presents the variations in plasma concentrations of leucine following the different dietary treatments. Table 4 reports the main effects (age, protein source, type of protein ingestion, and time) and their interactions. To resume, changes in plasma leucine concentrations were significantly higher in older subjects than in young subjects, following ingestion of whey protein than following ingestion of pea protein, and after ingestion of both protein solutions alone than after ingestion of both protein solutions following a standardized meal. Several significant interaction effects were observed, and one of them was the protein source × type of protein ingestion effect. This interaction effect underlined that the difference in plasma leucine concentration variations between taking the protein alone or after a standardized meal is significantly greater with whey protein than with pea protein. Baseline leucine concentrations were significantly lower in plasma from older participants than young participants (−18 µmol/L, age effect) (Table 5). The iCmax and iAUC of plasma leucine concentration variations were lower after ingesting the tested proteins in a standardized meal compared to alone (−24% and −27%, respectively; type of protein ingestion effect). The iCmax and iAUC of plasma leucine concentration variations were significantly higher after ingesting whey protein solution than pea protein solution in young and older subjects (+113% and +103%, respectively; protein source effect). There was a significant effect of type of protein ingestion × protein source interaction on iCmax and iAUC of plasma leucine concentration changes. Note that the iAUC of leucine concentration variations were higher in plasma from older participants than plasma from young participants (+15%; age effect).

#### 3.2.2. Arginine

There was no difference in baseline plasma arginine concentrations between young and older participants (Table 5). However, the iCmax, and iAUC of plasma arginine concentration changes were higher in response to ingestion of pea protein than in response to ingestion of whey protein (+117% and +173%, respectively; protein source effect: *p* < 0.001). There was a significant type of protein ingestion × protein source effect on the iCmax and iAUC of plasma arginine concentration changes.

#### 3.2.3. Sulfur-Containing Amino Acids (SCAA)

The baseline concentration, iCmax and iAUC of plasma SCAA concentration changes were significantly higher in older subjects than in young subjects (Table 5). As previously observed for leucine, the iCmax and iAUC of SCAA concentration changes were higher following consumption of whey protein solution compared to pea protein solution (+414% and +564%, respectively; protein source effect: *p* < 0.001), and there was an effect of age × protein source interaction on the iAUC of SCAA concentration changes (*p* < 0.05).

#### 3.2.4. Essential Amino Acids (EAA)

The baseline concentration of plasma EAA was significantly lower in older subjects than in young subjects (−108 µmol/L; age effect: *p* < 0.01), but the iAUC of plasma EAA concentration changes in response to dietary treatments was greater in older subjects than in young subjects (+13%; age effect: *p* < 0.05) (Table 5). There were significant main effects of the protein source and type of protein ingestion on the iCmax and iAUC of plasma EAA concentration changes. iAUC and iCmax values were greater following ingestion of whey protein and ingestion of both protein solutions alone (*p* < 0.001 for all).

#### 3.2.5. Non Essential Amino Acids (NEAA)

There was no age effect on baseline plasma NEAA concentrations or the iCmax or iAUC of plasma NEAA concentration changes (Table 5). Ingestion of pea protein induced higher iCmax and iAUC of plasma NEAA concentration variations than ingestion of whey protein (+10% and +15%, respectively; protein source effect: *p* < 0.05 and *p* < 0.01, respectively).

### 3.3. Changes in Plasma Glucose and Insulin Concentration

#### 3.3.1. Glucose

In accordance with data obtained from the screening visit, baseline plasma glucose concentrations tended to be lower in young participants than in older participants (age effect: *p* = 0.064) (Table 6). As expected, the Cmax and AUC of glucose concentration changes were higher after ingestion of protein solution following a standardized meal than after ingestion of protein solution alone (+24% and +7%, respectively; type of protein ingestion effect: *p* < 0.001 for all). There was no significant protein source effect on the Cmax or the AUC of glucose concentration changes. However, there was an effect of age × protein source interaction on the Cmax of glucose concentration changes (*p* < 0.01), which reflected the significant increase in Cmax after ingestion of pea protein following a standardized meal compared to ingestion of pea protein alone and the non-significant increase in Cmax after ingestion of whey protein following a standardized meal compared to ingestion of whey protein alone. We also found a significant effect of age × type of protein ingestion interaction on the AUC of plasma glucose concentration changes and a significant age × protein source × type of protein ingestion interaction on plasma glucose Cmax.

#### 3.3.2. Insulin

As a result of the plasma glucose concentration variations, the Cmax and AUC of plasma insulin concentration changes were greater after ingestion of protein solution following a standardized meal than after ingestion of protein solution alone (+226% and +229%, respectively; type of protein ingestion effect: *p* < 0.001 for all). Similar to glucose, there was no between-protein-source (pea vs. whey) difference in Cmax and AUC of plasma insulin concentration changes. There was a significant effect of age × protein source interaction on plasma insulin Cmax and AUC (*p* < 0.01). Note that the effect of the three-factor interaction (age × protein source × type of protein ingestion) on the AUC of plasma insulin concentration variations was significant (*p* < 0.05).

### 3.4. Treatment of C2C12 with Plasmas from the Human Dietary Study

In the results presented above, we reported that participant plasmas contained different concentrations of pro-anabolic factors, i.e., leucine, arginine, and insulin, and the anti-anabolic factor IL-6, depending on the main effects of age (young vs. older), protein source (pea protein vs. whey protein), type of protein ingestion (ingestion of protein solution alone vs. ingestion of protein solution after a standardized meal), and their interactions. To investigate the overall anabolic capacity of subjects’ plasmas, we conditioned C2C12 myotubes with plasmas from the human dietary study and then used western blotting and the SUnSET technique to measure the level of activation of p70 S6 kinase, an intermediate of the translation initiation step, and protein synthesis. 

**Figure 3:**
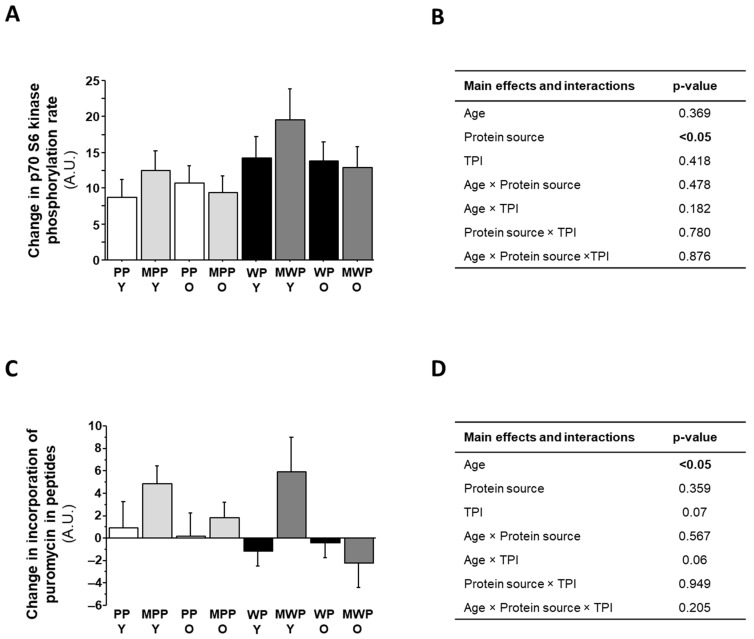
The p70 S6 kinase phosphorylation state and protein synthesis in C2C12 myotubes conditioned with media containing plasmas prepared from the human study. After 5 days of differentiation, C2C12 myotubes were treated with 10% human plasma in HBSS media for 4 h in the presence of 1 µM puromycin. We compared two plasma samples from each participant at each dietary treatment and calculated the difference between the two values, i.e., the value obtained with plasma samples at the peak plasma leucine concentration (Cmax) minus the value obtained with plasma samples prepared at the baseline timepoint (T0). The graphs plot differences for (**A**) the activation state of p70 S6 kinase and (**C**) the incorporation of puromycin in neosynthesized peptides. The tables display *p* values for the main effects and interactions following a 3-way repeated measures ANOVA for differences in (**B**) changes in the activation state of p70 S6 kinase and (**D**) changes in the incorporation of puromycin in neosynthesized peptides after the PP, WP, MPP, and MWP dietary treatments. Results are expressed as means ± SEM. Significant main effects or interactions are in bold (*p* < 0.05). PP: pea protein solution; WP: whey protein solution; MPP: standardized meal plus pea protein solution; MWP: standardized meal plus whey protein solution.

The p70 S6 kinase phosphorylation state measured in C2C12 myotubes treated with baseline human plasmas was significantly higher with plasmas from young participants compared with plasmas from older participants (96.7 ± 3.2 arbitrary units and 80.9 ± 3.4 arbitrary units, respectively; *p* < 0.05). The stimulation of p70 S6 kinase activation state in C2C12 myotubes, i.e., the change in p70 S6 kinase phosphorylation state between the value measured with plasmas at leucine Cmax minus the value measured with baseline plasmas, was greater when participants consumed whey protein solution than pea protein solution (+45%, *p* < 0.05; Figure 3A,B). There were no main effects on age, type of protein ingestion, or interaction effects. However, unlike the p70 S6 kinase activation state, protein synthesis, i.e., puromycin incorporation in new peptides, was similar in C2C12 cells treated with baseline plasma from young subjects and older subjects (102.8 ± 2.1 arbitrary units and 101.4 ± 2.0 arbitrary units, respectively, *p* = 0.601).

In contrast, the protein synthesis stimulation in C2C12 myotubes, which we measured as the increase of protein synthesis in cells treated with plasmas at leucine Cmax compared to cells treated with baseline plasmas, was significantly lower with plasma from older participants than young participants (−0.21 ± 0.89 arbitrary units and +2.60 ± 1.14 arbitrary units, respectively, *p* < 0.05; Figure 3C,D). There was no between-protein-source (pea vs. whey) difference in protein synthesis stimulation in C2C12 myotubes conditioned with participant plasmas. However, stimulation of protein synthesis tended to be higher following ingestion of protein after a standardized meal than following ingestion of protein solution alone (*p* = 0.07).

## 4. Discussion

In recent years, there has been growing interest in understanding the potential benefits of plant-based diets, particularly in the context of aging populations. However, in the present investigation, we showed that different types of dietary protein ingestion, e.g., whey proteins and pea proteins, are associated with specific amino acid profiles in young and older adults. In particular, the blood amino acid signature was markedly different in older participants compared to young participants after ingestion of proteins alone or in a complete meal.

Overall, the plasma concentrations of leucine, EAA, and SCAA measured after an overnight fast were different between young and older subjects. Blood concentrations of leucine and EAA were lower in older individuals, whereas blood concentrations of SAA were higher. Our observations on serum leucine and EAA are in line with previous studies [30,31]. For instance, a recent study comparing individual EAA responses between pea proteins and whey proteins showed higher peaks of EAA after a whey protein shake consumption compared to a pea protein shake consumption [32]. Even though amino acid levels can reflect long term dietary intake [33], the issue is around the extent to which serum leucine and EAA levels are influenced by other non-dietary factors such as insulin and low-grade inflammation, or inflamm’aging. Fasting blood levels of amino acids are, in general, in a steady state and reflect a balance between the rate of appearance and the rate of disappearance. Low blood concentrations of amino acids during fasting can result from a decrease in the rate of appearance due to low protein intake and/or a decrease in the mobilization of amino acid stores and/or changes in the rate of amino acid catabolism [34]. In obese people, Newgard et al. also identified a cluster of obesity-associated decreases in specific amino acids, including certain EAAs such as leucine [35]. The authors concluded that the deficiency in insulin-like growth factor (IGF-1) and increased inflammation found in the obese subjects force the circulating EAA pool to be diverted away from protein synthesis and into catabolic pathways. These changes may reflect an overload of amino acid catabolism in obese subjects [35]. As a reduction in IGF-1 production capacity and an increase in inflammation also occur during aging, we can put forward a similar hypothesis here. It has also been shown that lower blood concentrations of leucine and EAA in the elderly are associated with lower lean muscle mass [36]. Precisely, a previous study identified differences in muscle parameters between quartiles of serum EAA, with lower muscle mass in individuals with the lowest EAA blood concentrations measured in the fasting state [30]. Taken as a whole, a lower fasting blood concentration of leucine and EAA in older people may be the cumulative result of a lower dietary protein intake, a lower lean body mass, and metabolic abnormalities such as inflammation and lower production of anabolic hormones.

The lower fasting leucinemia found here in the older subjects may explain the lower activation of the p70 S6 kinase phosphorylation rate when the plasmas were applied to muscle cells in vitro. However, this difference does not translate into an age-related difference in capacity to stimulate protein synthesis.

Differences in SCAA metabolism have been found between older adults and young adults, with an approximately 50% slower glutathione synthesis rates in older subjects relative to their young counterparts [37]. This slower glutathione synthesis rate was significantly associated with higher concentrations of markers of oxidative stress. It may also explain the higher blood concentration of SCAA found in older subjects here, due to the lower use of these amino acids for the synthesis of glutathione, thus leading to a reduced rate of disappearance of SCAA in the liver. We also observed that blood SCAA concentration remained higher in older subjects than in young subjects after the consumption of animal- or plant-source proteins, whether alone or in a meal. Overall, results point to an age effect on the rate of disappearance of these amino acids, reflecting changes in their metabolism, whether for glutathione synthesis or for methylation pathways [37,38].

A major finding of this study was that postprandial blood concentrations of leucine and EAA were higher when dietary proteins were consumed alone than within a meal. This was the case for both the animal-source protein and the plant-source protein, despite their differences in amino acid composition, in particular leucine and EAA. There is little work comparing the incremental effects of ingesting protein alone or within a complete meal on aminoacidemia. Nevertheless, it is reasonable to hypothesize that the other elements of the meal and/or the metabolic response to the complete meal may modify the concentrations of amino acids found in the blood [39,40]. For example, in the presence of increased amino acid concentrations, insulin stimulates protein synthesis and inhibits protein degradation in several tissues, especially skeletal muscle, which accounts for almost half of the body’s proteins [41,42]. Therefore, as the body uses dietary amino acids to synthesize new proteins, there will be a lower postprandial elevation of amino acids, particularly EAA and leucine. The inhibition of tissue protein degradation by insulin also helps to reduce the rate of amino acid appearance in the bloodstream. Note that these variations were different between young and older people, i.e., the incremental increase in blood leucine concentration (leucine iAUC) was higher in older people after protein ingestion. If we stay with the hypothesis of metabolic regulation of amino acid fluxes by insulin, then the difference in insulin sensitivity between young and older individuals may explain why postprandial leucinemia remained at a higher level in older subjects. Note that here, the older participants tended to have higher fasting blood glucose and showed a greater rise in postprandial blood glucose concentration (glucose AUC), regardless of the protein contained in the meal. Taken together, these data point to a potential reduction in insulin sensitivity in older subjects. A reduction in insulin sensitivity may blunt its stimulating action on protein synthesis and reduce its inhibiting effect on protein degradation in insulin-dependent tissues [43]. This metabolic change would result in a lower slowdown of the rate of appearance and lower stimulation of the rate of disappearance in the elderly, and consequently a higher postprandial blood leucine concentration. Differences observed here in some inflammatory markers, e.g., IL6, may also explain this phenomenon, as inflammation contributes to insulin resistance and the dysregulation of protein metabolism [44]. This increase in catabolic factors such as proinflammatory cytokines, together with a decrease in pro-anabolic factors such as anabolic hormones, can also explain the lower effect of plasma from older subjects on the in vitro stimulation of protein synthesis in C2C12 myotubes. Meals containing proteins also stimulate glucagon secretion. It is believed that the purpose of the glucagon release is, by stimulating hepatic glucose production, to avert hypoglycemia resulting from the concomitant insulin secretion [45]. Glucagon’s main effect on blood amino acids is thus to increase the consumption of glucogenic amino acids, presumably for gluconeogenesis. As a consequence, glucagon has been reported to increase the disposal of amino acids after protein or amino acid intake [46]. It is likely that, through this diversion of amino acids, glucagon diminishes the precursor pool of amino acids available for protein synthesis, thus limiting the insulin- and amino acid-induced stimulation of protein synthesis [47]. Direct evidence for and the mechanism of this reported effect have not yet been elucidated.

Variations in leucinemia and SCAA were greater after the ingestion of whey proteins compared to pea proteins, mainly due to the difference in leucine and SCAA contents between these two proteins. Despite this difference in postprandial leucine and EAA concentrations between the animal protein and plant protein and the associated stronger activation of the P70 phosphorylation level by whey protein, it is interesting to note that the stimulating effect of plasmas on the rate of protein synthesis in myotubes was not different between whey and pea protein. This result may show that these two proteins share the same ability to regulate muscle protein metabolism when provided under the same conditions, in which case these pea proteins could be used by the elderly to improve postprandial anabolism. Note that the variations in postprandial insulinemia found here were not age-dependent. The anabolic effects of insulin on C2C12 cells can therefore operate in the same way regardless of the age of the subjects providing the plasma, as the cells used in vitro have the same sensitivity to insulin. This would possibly not have been the case if we had tested the effects of these conditioned media on cells in which insulin sensitivity was matched to the age of the subjects.

The effect of the high level of plasma leucine following the ingestion of whey protein on the mTOR pathway and its repercussions on protein synthesis could be compensated by the greater content of other protein synthesis-regulating amino acids, such as arginine, in plasmas sampled after the ingestion of pea proteins [48]. Among the specific modifications of certain amino acids, we noted a greater increase in peak and incremental concentrations of blood arginine after the consumption of pea proteins compared to whey proteins, regardless of the conditions, i.e., alone or part of a complete meal. These differences are firmly explained by the differences in arginine contents of the two proteins (3.5 times more in pea protein). Note too that this intake makes it possible to double or even triple blood arginine concentrations in the elderly, despite a higher splanchnic extraction of dietary amino acids in this population [5]. Greater arginine intake could have great benefits for aging subjects. Studies have shown that arginine intake in older people leads to a significant increase in serum IGF-1 concentrations, positive nitrogen balance, and a decrease in total serum cholesterol with a reduction in low-density lipoprotein [49]. Several human and experimental animal studies have also indicated that arginine intake has multiple beneficial effects over the course of aging, including reducing the risk for vascular and heart diseases and improving immune response [50]. Taken together, these data suggest that consumption of dietary proteins containing a high arginine content, such as pea proteins, may be beneficial for older adults. Furthermore, as conducted here, another in vitro study investigated the effect of arginine on protein synthesis in differentiated mouse C2C12 myoblasts by labeling the newly synthesized polypeptides with low concentrations of puromycin [51]. The C2C12 cells were supplied with extra arginine in the culture media, and the arginine increased the rate of protein synthesis in a nitric oxide (NO)-dependent manner [51]. These results could explain why we did not observe any difference between pea proteins and whey proteins in terms of the capacity of conditioned plasma to stimulate muscle protein synthesis, despite the lower leucine content of pea proteins. The high arginine content of pea protein could compensate for its lower leucine content and help activate the NO-dependent anabolic pathways within the muscle cell. This observation is confirmed by the stronger stimulation of P70 S6 kinase with culture media conditioned with plasma stemming from the consumption of whey protein, which contains a higher content of leucine, compared to plasma stemming from the consumption of pea protein, which contains less leucine but more arginine. Interestingly, we did not observe any differences between the effects of plasmas sampled after either whey or pea protein ingestion on protein synthesis in myotubes in young but also in older subjects, regardless of the conditions, i.e., whether the protein was given alone or within a complete meal. This result shows that pea protein, such as whey protein, could be able to activate muscle protein synthesis in aged muscle. Similar findings have already been reported in old rats [52,53] and in other populations, such as trained individuals [18,54,55].

A limitation of this work is that we did not measure the lean body mass and muscle mass of the individuals. By measuring such parameters, it would have been possible to express protein intake regarding lean body mass and not regarding body weight, as lean body mass decreases with age. Additionally, we could have discussed the rate of appearance and the rate of disappearance of amino acids in plasma regarding the amount of lean mass. Finally, we could have interpreted the results obtained concerning the rate of protein synthesis within C2C12 cells treated with plasmas regarding the muscle mass of the individuals, in particular young versus older subjects. Compounds limiting muscle protein synthesis could be present in the plasma of individuals with the lowest muscle masses and could explain the differences observed in C2C12 cells.

## 5. Conclusions

In conclusion, this study used the relatively novel conditioned media method to test the anabolic action on muscle cells of a particular plasma aminoacidemia obtained after ingestion of a specific protein. This approach can potentially produce new data to redefine the quality of a dietary protein. Overall, we show that although the postprandial variations of blood amino acids and meal effectors are different between young and older individuals, plasmas from these young and older subjects have little difference in effect on protein synthesis in myotubes in vitro. The aging-related alterations in muscle response to certain amino acids, such as leucine, due to an increase in splanchnic extraction of dietary leucine and a loss of muscle leucine sensitivity, might be compensated by the ingestion of other amino acids that have been shown to be effective during aging, such as arginine. These observations highlight the growing interest in proteins containing high levels of this kind of amino acid, such as pea proteins, to optimize nutritional intake and support healthy aging trajectories in older people who are typically exposed to dysregulated protein metabolism, loss of skeletal muscle, and ultimately sarcopenia. These plant proteins also need to be highly digestible. Pea protein isolates have equivalent digestibility to whey protein and are therefore good candidates for plant protein-based optimization of nutritional intake, even in older subjects. We can therefore postulate that, considering the outcomes determined in the present study, pea and whey proteins have the same qualities in terms of their properties to maintain muscle protein synthesis. It can therefore be recommended that older people who do not consume enough protein from animal sources increase their consumption of this type of plant protein.

## Figures and Tables

**Table 1:** Composition and amino acid content of the protein sources.

	Pronativ^®^ 95	Nutralys^®^S85
Composition (g/100 g powder)		
Moisture	6.2	6.0
Proteins	90.2	80.2
Carbohydrates	0.2	0.4
Fat	0.3	9.1
Amino acid content (g/100 g protein)		
Alanine	4.7	4.3
Arginine	2.4	8.5
Aspartic acid	11.2	11.6
Cystine	2.8	1
Glutamic acid	16.9	17.5
Glycine	1.8	4.1
Histidine	2.0	2.4
Isoleucine	5.4	4.8
Leucine	11.9	8.4
Lysine	9.6	7.5
Méthionine	2.1	1
Phenylalanine	3.5	5.4
Proline	4.5	4.5
Serine	4.3	5.2
Threonine	4.9	3.9
Tryptophan	2.2	0.8
Tyrosine	3.7	4.0
Valine	5.0	5.2

**Table 2:** Baseline physical and biochemical characteristics of the study participants in a fasted state.

	Young (*n* = 15)	Older (*n* = 15)	*p*-Value
Physical characteristics			
Age (years)	23.8 ± 0.7	70.5 ± 1.2	<0.001
Weight (kg)	79.7 ± 2.4	73.1 ± 2.1	<0.05
Height (m)	1.78 ± 0.02	1.71 ± 0.01	<0.001
BMI (kg/m^2^)	24.9 ± 0.4	25.0 ± 0.5	
Biochemical characteristics			
Glucose (g/L)	0.792 ± 0.022	0.867 ± 0.023	<0.05
Cholesterol (mmol/L)	4.22 ± 0.21	5.62 ± 0.16	<0.001
Triglycerides (g/L)	0.90 ± 0.08	1.03 ± 0.09	

Results are expressed as means ± SEM. Only *p* values that were lower than 0.05 are reported.

**Table 3:** Plasma inflammation markers of the study participants before each dietary treatment.

	Young (*n* = 15)	Old (*n* = 15)	*p*-Value
TNFα (pg/mL)	0.864 ± 0.056	0.985 ± 0.101	
IL-6 (pg/mL)	1.215 ± 0.167	2.789 ± 0.612	<0.05

Results are expressed as means ± SEM. Only *p* values that were lower than 0.05 are reported.

**Table 4:** *p*-value table of main effects and interactions following a 4-way repeated measures ANOVA for assessing differences in plasma leucine concentration changes.

Main Effects and Interactions	*p*-Value
Age	**<0.001**
Protein source	**<0.001**
TPI	**<0.001**
Time	**<0.001**
Age × Protein source	0.611
Age × TPI	0.213
Age × Time	**<0.05**
Protein source × TPI	**<0.05**
Protein source × Time	**<0.001**
TPI × Time	**<0.001**
Age × Protein Source × TPI	0.502
Age × Protein source × Time	0.500
Age × TPI × Time	0.588
Protein source × TPI × Time	**<0.05**
Age × Protein source × TPI × Time	0.818

A 4-way repeated measures ANOVA was performed to analyze differences in plasma leucine concentration changes following PP, WP, MPP, and MWP dietary treatments. Significant main effects or interactions are bold (*p* < 0.05). TPI: type of protein ingestion.

**Table 5:** Plasma amino acid concentration changes.

	Young				Older				Main Effects and Interactions Value						
	PP	WP	MPP	MWP	PP	WP	MPP	MWP	Age	Protein Source	TPI	Age × Protein Source	Age × TPI	Protein Source × TPI	Age × Protein Source × TPI
**Leucine**															
Baseline (µmol/L)	159 ± 6 ^ac^	150 ± 7 ^ab^	151 ± 6 ^ac^	157 ± 7 ^ac^	133 ± 4 ^b^	142 ± 11 ^bc^	131 ± 3 ^b^	140 ± 8 ^bc^	**<0.05**	-	-	-	-	-	-
iCmax (µmol/L)	187 ± 15	395 ± 17	149 ± 11	329 ± 23	217 ± 11	464 ± 23	157 ± 13	326 ± 25	0.152	**<0.001**	**<0.001**	0.491	0.07	**<0.01**	0.161
iAUC (µMol/L × 180 min)	19,771 ± 1242	39,547 ± 1969	13,546 ± 923	31,259 ± 1568	24,125 ± 1025	46,149 ± 1795	16,511 ± 1303	33,251 ± 2057	**<0.05**	**<0.001**	**<0.001**	0.723	0.183	**<0.05**	0.328
**Arginine**															
Baseline (µmol/L)	93 ± 4	84 ± 4	91 ± 6	90 ± 5	95 ± 5	94 ± 5	94 ± 5	96 ± 5	0.315	-	-	-	-	-	-
iCmax (µmol/L)	151 ± 17	66 ± 3	120 ± 11	57 ± 5	165 ± 7	75 ± 4	124 ± 10	60 ± 3	0.354	**<0.001**	**<0.001**	0.793	0.494	**<0.05**	0.828
iAUC (µMol/L × 180 min)	15,791 ± 1263	6385 ± 335	12,033 ± 940	4083 ± 367	17,294 ± 862	6680 ± 588	13,268 ± 929	4187 ± 359	0.289	**<0.001**	**<0.001**	0.342	0.787	**<0.05**	0.958
**SCAA**															
Baseline (µmol/L)	89 ± 2 ^a^	85 ± 2 ^a^	89 ± 2 ^a^	89 ± 2 ^a^	105 ± 2 ^b^	104 ± 3 ^b^	104 ± 3 ^b^	104 ± 2 ^b^	**<0.001**	-	-	-	-	-	-
iCmax (µmol/L)	10 ± 2	58 ± 2	10 ± 1	56 ± 4	15 ± 3	76 ± 5	14 ± 2	65 ± 5	**<0.01**	**<0.001**	0.090	0.058	0.280	0.175	0.353
iAUC (µMol/L × 180 min)	567 ± 132	4157 ± 159	550 ± 114	4055 ± 351	743 ± 116	5397 ± 425	928 ± 248	4896 ± 386	**<0.01**	**<0.001**	0.600	**<0.05**	0.812	0.264	0.382
**EAA**															
Baseline (µmol/L)	1072 ± 34 ^a^	1002 ± 34 ^ac^	1048 ± 33 ^ab^	1050 ± 40 ^ac^	926 ± 22 ^b^	955 ± 50 ^b^	909 ± 19 ^bc^	948 ± 35 ^bc^	**<0.01**	-	-	-	-	-	-
iCmax (µmol/L)	822 ± 65	1358 ± 55	720 ± 50	1200 ± 78	967 ± 44	1594 ± 78	750 ± 57	1200 ± 87	0.139	**<0.001**	**<0.001**	0.662	0.07	0.05	0.300
iAUC (µMol/L × 180 min)	89,090 ± 5122	137,753 ± 6133	71,382 ± 4080	116,165 ± 5676	105,487 ± 4197	157,004 ± 6764	82,356 ± 6037	123,419 ± 7617	**<0.05**	**<0.001**	**<0.001**	0.944	0.308	0.232	0.580
**NEAA**															
Baseline (µmol/L)	1846 ± 58	1721 ± 64	1809 ± 73	1738 ± 59	1679 ± 48	1712 ± 64	1654 ± 42	1694 ± 48	0.184	-	-	-	-	-	-
iCmax (µmol/L)	754 ± 66	678 ± 31	787 ± 63	733 ± 57	869 ± 34	786 ± 48	846 ± 76	763 ± 52	0.163	**<0.05**	0.793	0.770	0.405	0.839	0.850
iAUC (µMol/L × 180 min)	71,350 ± 4648	63,688 ± 4158	80,023 ± 6328	71,868 ± 6841	79,100 ± 4742	68,556 ± 6925	90,418 ± 9146	75,043 ± 7353	0.301	**<0.01**	0.08	0.478	0.961	0.670	0.729

Results are expressed as means ± SEM. a ≠ b ≠ c with *p* < 0.05. TPI: type of protein ingestion. iCmax: incremental maximal plasma concentration of an amino acid or a sum of several amino acids. iAUC: incremental area under the curve. SCAA: sulfur-containing amino acids (i.e., cysteine and methionine). EAA: essential amino acids. NEAA: non essential amino acids. PP: pea protein solution. WP: whey protein solution. MPP: standardized meal plus pea protein solution. MWP: standardized meal plus whey protein solution. Significant main effects or interactions are in bold (*p* < 0.05).

**Table 6:** Plasma glucose and insulin concentration changes.

	Young				Older				Main Effects and Interactions Value						
	PP	WP	MPP	MWP	PP	WP	MPP	MWP	Age	Protein Source	TPI	Age × Protein Source	Age × TPI	Protein Source × TPI	Age × Protein Source × TPI
**Glucose**															
Baseline (g/L)	0.940 ± 0.015	0.938 ± 0.019	0.920 ± 0.014	0.863 ± 0.027	0.965 ± 0.022	0.966 ± 0.0.017	0.933 ± 0.018	0.960 ± 0.022	0.064	-	-	-	-	-	-
Cmax (g/L)	0.977 ± 0.015 ^d^	0.993 ± 0.018 ^d^	1.169 ± 0.034 ^abcd^	1.237 ± 0.040 ^abc^	1.017 ± 0.023 ^cd^	1.042 ± 0.031 ^bcd^	1.347 ± 0.058 ^a^	1.248 ± 0.047 ^ab^	0.098	0.861	**<0.001**	**<0.01**	0.254	0.333	**<0.05**
AUC (g/L × 360 min)	162.2 ± 2.7	161.8 ± 2.5	170.7 ± 5.1	166.5 ± 4.4	170.7 ± 3.8	172.2 ± 3.9	193.3 ± 7.1	186.3 ± 6.9	**<0.05**	0.179	**<0.001**	0.907	**<0.05**	0.070	0.488
**Insulin**															
Baseline (µiU/mL)	6.44 ± 1.03	5.57 ± 0.96	4.56 ± 0.98	5.03 ± 0.62	4.07 ± 0.69	4.01 ± 0.66	4.10 ± 0.80	4.78 ± 0.82	0.181	-	-	-	-	-	-
Cmax (µiU/mL)	18.80 ± 2.11	25.3 ± 2.1	62.4 ± 6.5	82.0 ± 8.3	17.2 ± 1.9	20.6 ± 1.9	65.5 ± 11.7	57.0 ± 6.2	0.240	0.100	**<0.001**	**<0.05**	0.410	0.929	0.067
AUC (µiU/mL × 360 min)	2253 ± 170 ^a^	2657 ± 249 ^a^	6825 ± 548 ^b^	8194 ± 729 ^b^	1998 ± 266 ^a^	2249 ± 289 ^a^	7760 ± 995 ^b^	7322 ± 783 ^b^	0.819	0.107	**<0.001**	**<0.05**	0.646	0.720	**<0.05**

Results are expressed as means ± SEM. a ≠ b ≠ c ≠ d with *p* < 0.05. TPI: type of protein ingestion. Cmax: maximal plasma concentration of an amino acid or a sum of several amino acids. AUC: area under the curve. PP: pea protein solution. WP: whey protein solution. MPP: standardized meal plus pea protein solution. MWP: standardized meal plus whey protein solution. Significant main effects or interactions are in bold (*p* < 0.05).

## Data Availability

Data is unavailable due to privacy reasons.

## Supplementary Materials

The following supporting information can be downloaded at: https://www.mdpi.com/article/10.3390/nu16172870/s1, Table S1: Composition of the test protein solutions; Table S2: Composition of the standardized meals.
