# Supplementary material for: Circulating Amino Acid Concentration after the Consumption of Pea or Whey Proteins in Young and Older Adults Affects Protein Synthesis in C2C12 Myotubes
Source: Nutrients. 2024 Aug 27;16(17):2870. doi: 10.3390/nu16172870 (PMC11397729; doi:10.3390/nu16172870)
Supplement: Supplementary file 1 [file nutrients-16-02870-s001.zip › nutrients-3089837-supplementary.pdf]

Supplementary Table S1

Composition of the test protein solutions.

Food	Whey protein solution	Pea protein solution
Test protein source		
Pronativ®95 (g/kg of body weight)	0.46	-
NUTRALYS®S85 Plus (g/kg of body weight)	-	0.52
Water (mL)	300	300

Supplementary Table S2

Composition of the standardized meals.

Food	Standardized meal associated with WP	Standardized meal associated with PP
**g/kg of body weight**		
Food		
Red beetroot	1.11	1.11
Salad dressing	0.22	0.22
Mashed potatoes	4.91	4.99
Butter	0.33	0.28
Fruit compote	1.11	1.11

WP, test whey protein solution.

PP, test pea protein solution.
